# Etiopathogenesis, Challenges and Remedies Associated With Female Genital Tuberculosis: Potential Role of Nuclear Receptors

**DOI:** 10.3389/fimmu.2020.02161

**Published:** 2020-10-15

**Authors:** Shalini Gupta, Pawan Gupta

**Affiliations:** Department of Molecular Biology, CSIR-Institute of Microbial Technology, Chandigarh, India

**Keywords:** nuclear receptors, uterine receptivity, cytokine modulation, female genital tuberculosis, recurrent implantation failure, endometrium regeneration, extrapulmonary tuberculosis

## Abstract

Extra-pulmonary tuberculosis (EPTB) is recognized mainly as a secondary manifestation of a primary tuberculosis (TB) infection in the lungs contributing to a high incidence of morbidity and mortality. The TB bacilli upon reactivation maneuver from the primary site disseminating to other organs. Diagnosis and treatment of EPTB remains challenging due to the abstruse positioning of the infected organs and the associated invasiveness of sample acquisition as well as misdiagnosis, associated comorbidities, and the inadequacy of biomarkers. Female genital tuberculosis (FGTB) represents the most perilous form of EPTB leading to poor uterine receptivity (UR), recurrent implantation failure and infertility in females. Although the number of TB cases is reducing, FGTB cases are not getting enough attention because of a lack of clinical awareness, nonspecific symptoms, and inappropriate diagnostic measures. This review provides an overview for EPTB, particularly FGTB diagnostics and treatment challenges. We emphasize the need for new therapeutics and highlight the need for the exaction of biomarkers as a point of care diagnostic. Nuclear receptors have reported role in maintaining UR, immune modulation, and TB modulation; therefore, we postulate their role as a therapeutic drug target and biomarker that should be explored in FGTB.

## Introduction

*Mycobacterium tuberculosis (M. tuberculosis)* is an etiological agent that causes tuberculosis (TB), which is a health issue of global importance. TB profoundly exists in two forms, i.e., pulmonary and extrapulmonary. The most prevalent site of TB infection is the lungs; this is called pulmonary TB (PTB), where the bacilli are phagocytosed in alveolar macrophages and are contagious *via* aerosol dissemination. TB bacilli can also disseminate to other organs and causes extrapulmonary tuberculosis (EPTB). The genital organs are also an important site for dissemination. **Table 1** shows the distribution of TB cases at extrapulmonary sites (1). EPTB is mainly considered to be a secondary manifestation of the primary infection, which is rarely contagious; however, extrapulmonary involvement can occur with or without PTB. The World Health Organization (WHO) reported 7 million TB cases in 2018 of which 15% were EPTB (2). Additionally, approximately, 10%–50% of EPTB cases are reported to also have pulmonary manifestation (3). The prevalence of EPTB significantly contributes to TB-related morbidity and mortality and is a leading cause of maternal mortality. In a case study, the highest mortality rates are reported for meningitis TB (9.6%) and peritoneal TB (8.5%) (4). Peritoneal TB and female genital TB (FGTB) are a threat to human species propagation (5). Bacterial dissemination leading to EPTB occurs majorly *via* three different channels, i.e., hematogenous, lymphatic, and direct spread (6). Additionally, producing new blood vessels through vascular endothelial growth factor (VEGF) can assist in bacterial dissemination (7). Some rare modes of transmission include congenital transmission, accidental inoculation, therapeutic instillation, and vaccination (8). The atypical presentation, paucibacillary nature, arduousness in procuring appropriate clinical sample, lack of awareness among clinicians, and poor sensitivity of conventional microbiological techniques in EPTB, particularly FGTB, are challenges in diagnosis that further raise the cost due to disability. EPTB cases are on the rise; however, there is still a very extensive awareness gap compared to PTB (15% vs. 86%) (9). The aim of the WHO’s “end TB strategy” highlights the need for patient TB care and awareness programs in PTB (10). However, the information on EPTB needs to be adequately addressed. FGTB, which represents the most perilous form of EPTB, is steadily rising as one of the major causes of infertility in females. Globally, about 5%–10% of infertile women are reported to have FGTB (11). FGTB demands immediate attention because of its low recovery rates and the increased abortion rates observed during recent years. Primary infection of TB in the genital tract of females, albeit rare, may occur if the partner has active genitourinary TB. Despite our current understanding, it is vital that research into EPTB and especially FGTB is increased as it is critical to enhance our knowledge of this disease in order to effectively combat it.

**Table 1:** The bacterial manifestation reported at the surplus site and the prepotency.

Extrapulmonary forms	Occupied site of EPTB (%)	Mode of spread
Lymph node TB	35%	Direct
Pleural TB	20%	Hematogenous
Meningitis TB	5%	Hematogenous
Abdomen TB	3%	Direct
Miliary TB	8%	Hematogenous
Bone and joint TB	10%	Hematogenous
Genitourinary TB	9%	Hematogenous
Others	10%	

This review highlights the major challenges of EPTB, especially FGTB, and necessitates the need for research efforts for effective biomarker discovery in FGTB. The objective of this review is to introduce the diagnostic, treatment, and comorbidity challenges associated with EPTB and, in particular, FGTB and to raise fundamental biological questions regarding the impact of FGTB on female fertility and on the major issues of endometrium regeneration (ER), uterine receptivity (UR), and cytokine modulation (CM). This review covers the current knowledge of nuclear receptors (NRs), reported in regeneration, female reproduction, and in the maintenance of pregnancy with the aim of conceptually postulating that NRs should be explored in the diagnosis and combating of FGTB-associated female infertility.

## Epidemiology and Clinical Presentation of FGTB: The Silent Rise

FGTB is the most enigmatic form of EPTB, representing 15%–20% of EPTB cases (12, 13), and is responsible for poor UR, poor endometrial adhesions, and recurrent implantation failure (RIF) in females (14). However, the exact proportion of FGTB is not known due to underreporting of cases, nonspecific symptoms, misleading clinical appearance, and lack of diagnostic measures. Additionally, in a case study, approximately 75.6% of patients’ cases evaluated for infertility were diagnosed with FGTB (15). It is highly concerning because the manifestations are asymptomatic, and by the time FGTB is diagnosed, it has already left an impact on female fertility and morbidity. There is also a social stigma attached to FGTB that causes it to be difficult for women to talk openly about it. FGTB is known to mainly cause primary infertility rather than secondary infertility (16); therefore, even after successful treatment, conception rates are very low (19.2%), the success of pregnancy is very low (16.6%), and the birth rate is also extremely low (7.2%) (17, 18). The TB bacilli break out from the primary site of infection and reach the genital area generally through hematogenous spread (19). The most prevalent site of bacterial infection for FGTB includes the endometrium (50%–60%), fallopian tubes (95%–100%), ovaries (20%–30%), cervix (5%–15%), myometrium (2.5%), and vagina/vulva (1%) (19, 20). FGTB causes caseation, adhesions, ulcerations, and complete distortion of the cavity causing Asherman syndrome. The clinical appearance of FGTB is generally called “the considerable pretender” because it mimics ovarian carcinoma (21). FGTB represent various clinical symptoms of infertility (43%–74%), oligomenorrhea (54%), amenorrhea (14%), dysmenorrhea (12%–30%), abdominal pain (42.5%), menorrhagia (19%), dyspareunia (5%–12%), and postmenopausal bleeding (2%) (19, 22–25). The abovementioned clinical presentations arise because the ER capability is compromised, which contributes to recurrent pregnancy loss and infertility (**Table 2**). All these symptoms pertain to the endometrium, and its regeneration needs to be addressed and investigated. The key factors that modulate and exacerbate FGTB need to be identified.

**Table 2:** Various forms of clinical presentations of FGTB are shown along with signs and symptoms.

Extrapulmonary infection	Clinical presentation	Signs and Symptoms
TB of endometrium	Uterine leiomyoma	Pyometra
	Postmenopausal TB	Irregular vaginal bleeding and persistent leucorrhoea
	Oligomenorrhoea	Menstrual disturbance
	Amenorrhoea	Menstrual disturbance
	Menorrhagia	Abnormal vaginal discharge
TB of cervix	Ovarian carcinoma	Postcoital bleeding
TB of vulva	Tumor	Bloodstained discharge
TB of ovary	Perioophoritis	Tubo-ovarian masses
TB of fallopian tube	Salpingitis and tubal block	Ectopic pregnancy
	Infertility	Implantation failure
TB of pelvic	Fistula formation	Rupture of a tuberculous pyosalpinx
	Malaise	Pelvic inflammatory disease

## The Diagnostic Challenges of EPTB With an Emphasis on FGTB

**Figure 1:**
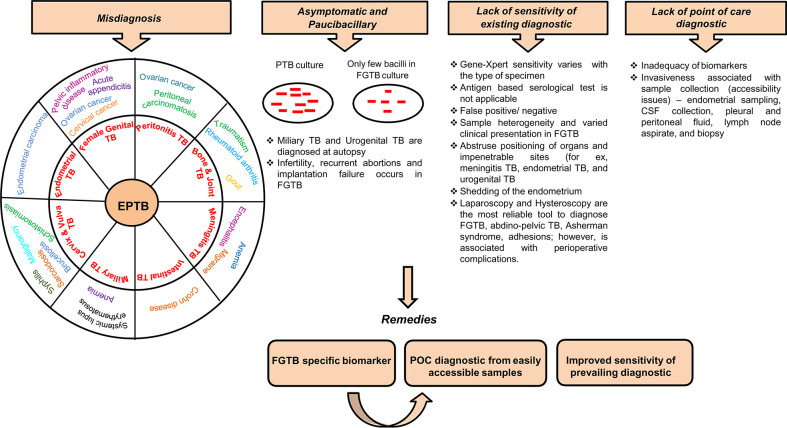
Diagnostic challenges and remedies for EPTB, in particular FGTB. Various challenges associated with EPTB diagnosis, such as misdiagnosis, often asymptomatic and paucibacillary nature of bacilli, lack of sensitivity of existing conventional methods, and lack of point-of-care diagnostics, lead to loss due to disability.

The diagnostic tools for EPTB include the nucleic acid amplification test (Gene-Xpert), immunological test, biopsy, body fluid examination, and sputum acid-fast bacillus (AFB) smear. Gene-Xpert shows high sensitivity in EPTB samples but is less in cerebrospinal fluid (CSF), i.e., 29% (26). The antibody-based serological test has poor sensitivity and is not applicable to EPTB samples (27). Blood transcriptomic biomarkers are identified in TB, which can easily discriminate between healthy and infected persons (28–31). The onset of TB can be predicted through metabolite changes in blood (32). Blood transcriptomic and metabolic signatures have improved diagnosis in TB and are being explored as probable diagnosis for EPTB (8, 33, 34). Systematic reviews on TB biomarkers, including antibodies, cytokines, chemokines, proteins, and metabolic activity markers have already been published (35). These biomarkers, to some extent, have also been studied in EPTB (36, 37). EPTB is largely undiagnosed in patients, especially when visceral sites are involved. The detection of EPTB, particularly FGTB, poses a major challenge with conventional methods. EPTB diagnosis is challenging because of misdiagnosis, arduousness in acquiring of clinical samples, being asymptomatic, and poor sensitivity of existing diagnostic (**Figure 1**). Generally, miliary TB is misdiagnosed with systemic lupus erythematosus (SLE) (38). EPTB, particularly peritoneal TB, may also be misinterpreted as ovarian cancer and peritoneal carcinomatosis (5, 39). Intestinal TB is misdiagnosed with Crohn disease (40). Bone and joint TB are misdiagnosed as rheumatoid arthritis, traumatism, and gout. Vulva and vaginal TB is misdiagnosed with malignancy (41). Invasiveness and constraints in obtaining biopsies prevent the early diagnosis of EPTB, and in addition, these diagnostic tests can cause incidental damages and infection; for instance, in the case of meningitis TB, extraction of CSF can possibly harm the nerves around the site of insertion. Biopsy, endoscopy, cystoscopy, and lumbar puncture are all performed depending on the case for other EPTBs (8). Meningitis TB is suspected when the patient is diagnosed with mental disturbance or is found to have lymphocytic pleocytosis (42). Due to the nonparticular symptoms, miliary TB and urogenital TB are often diagnosed at an autopsy (8, 43–46).

Being a paucibacillary disease, the diagnostic measures of FGTB involve a combination of bacteriological confirmatory measures. FGTB patients exhibit features of dysfunction of genital organs rather than any symptoms of infection. Repeatedly invasive techniques are utilized to acquire sufficient samples of body fluids, tissues, or biopsies. FGTB diagnosis is mainly done through endometrial samples using microscopy (AFB), histopathological detection of epithelioid granuloma on biopsy, and Gene-Xpert (41). Peritoneal fluid or biopsy for culture, endoscopy, and cervical cytology are also performed for diagnosis. However, histopathological findings are not specific for FGTB because of shedding of the endometrium. Magnetic resonance imaging and positron emission tomography have been used for detecting tubo-ovarian masses (47, 48). Loop-mediated isothermal amplification is the most convenient technique used for diagnosing FGTB (49). A laparoscopy combined with hysteroscopy is the most reliable tool to diagnose FGTB; however, this is associated with perioperative complications. Laparoscopy is risky because of the presence of many adhesions, which cover the pelvic organs and may hinder the diagnosis and can increase the risk of bleeding (41, 50). Hysteroscopy is associated with various complications, such as excessive bleeding, perforation, inability to distinguish and distend cavity, and flare-up of genital TB, which can cause abortions and infertility (51). FGTB, specifically endometrial TB, represents ulceration, caseous necrosis, and hemorrhage; this necessitates careful macroscopic sampling (51, 52). FGTB is a silent disease; rarely, it presents as abdominal pain, abnormal genital bleeding, and dyspareunia (53). The misdiagnosis rate is very high among FGTB patients and is associated with several complications. The disease is mistaken for other gynecological conditions or malignancy; for example, FGTB is misdiagnosed as ovarian cancer or chocolate cyst or pelvic inflammatory disease (PID) (54), and FGTB patients who are reported to have cervical TB may masquerade as cervical cancer (41, 55). Additionally, FGTB patients may be mistaken or coexist with acute appendicitis or ectopic pregnancy (52). TB of the vulva and cervix is very arduous to distinguish as it appears as brucellosis, schistosomiasis, tularemia, cervical amoebiasis, sarcoidosis, syphilis, or chancroid (56). Furthermore, a high level of drug resistance is witnessed in FGTB (57). Given the above challenges with FGTB diagnosis, including exceptional positioning of organs, associated invasiveness of sample collections, misdiagnosis, being asymptomatic, poor sensitivity, the emergence of drug resistance, and the lack of point of care, there is a strong need to identify FGTB-specific biomarkers. The biosignatures emanating from the pathogen have been reported for FGTB diagnosis (58). However, the sensitivity of detection in FGTB patient samples is very low because the infected sites are missed due to the paucibacillary nature of *M. tuberculosis*. We are focusing on the host-derived biomarkers for the prompt and accurate diagnosis of FGTB from easily accessible samples without utilizing any invasive procedure.

## Treatment Challenges of EPTB With an Emphasis on FGTB

**Figure 2:**
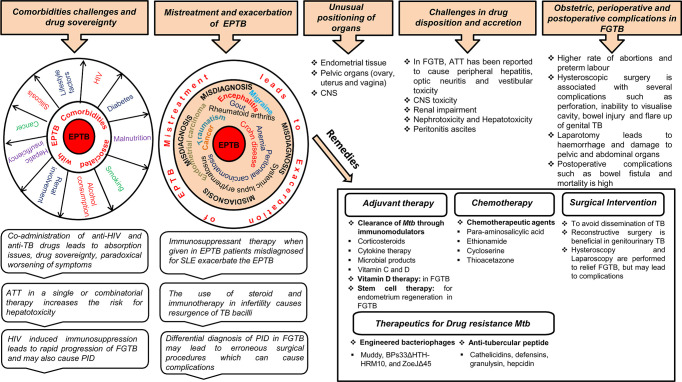
Treatment challenges and remedies for EPTB, in particular, FGTB. Various comorbidity challenges associated with EPTB are depicted. The risk of EPTB, particularly FGTB, occurrence increases in a comorbid condition depending on the severity of immunosuppression associated with these diseases. Coadministration of drugs results in drug sovereignty, toxicity, absorption issues, and paradoxical reactions in the body, which can further exacerbate the condition. EPTB misdiagnosis and subsequent mistreatment suppress the immune system to such an extent that it increases the bacterial prepotency of spreading to other organs (example genital organs) and exacerbation. Differential diagnosis in FGTB leads to erroneous surgical procedures, which can cause complications. ATT treatment in EPTB, in particular FGTB, faces drug disposition and accretion challenges. The unusual positioning of infected organs in EPTB illustrate treatment challenges, especially in meningitis TB, ovarian TB, urogenital TB, and endometrial TB. Due to the inaccessibility of organs in FGTB, surgical interventions are required to avoid dissemination of *M. tuberculosis*; however, several perioperative complications have been observed during surgery. Erroneous surgical procedures and mistreatment lead to obstetric and postoperative complications. Stem cell therapy, chemotherapy, vitamin D therapy, and surgical interventions can be beneficial in FGTB, whereas adjuvant therapy is known to be effective in EPTB, and engineered bacteriophages and antitubercular peptides are used for drug-resistant TB.

Treatment of EPTB faces major challenges from comorbidities (e.g., HIV coinfection or renal failure), drug sovereignty, misdiagnosis, drug disposition, and unusual positioning of a few organs, i.e., endometrium, central nervous system (CNS) (**Figure 2**). Chronic renal failure exacerbates EPTB more than TB (59). During renal impairment, DOTS therapy is eliminated by nonrenal routes; for example, by biliary secretion or through metabolism. Coadministration of anti-HIV and anti-TB drugs in a comorbid condition leads to absorption issues due to a reduction in the assimilation of the two key anti-TB drugs (rifampin and isoniazid) (60). Likewise, TB drugs also lower the levels of antiretroviral drugs; as soon as the antiretroviral therapy is initiated, it paradoxically results in worsening of symptoms or causes immune reconstitution inflammatory syndrome (1, 61). A high proportion of drug-induced liver injuries are observed in cirrhosis patients coinfected with TB (62). Ascites formed in the body in peritonitis TB present a problem for anti-TB drug disposition (63). Approximately 10%–20% of patients consuming ATT (anti-TB drugs; Ethambutol, Pyrazinamide, Isoniazid, and Rifampicin) either in a single or combinatorial therapy are at a risk of evolving hepatotoxicity (64–66). When EPTB is misdiagnosed as another disease, the treatment for the erroneous disease may exacerbate EPTB; for example, immunosuppressant therapy given when EPTB is misdiagnosed as chronic kidney disease exacerbates the actual case of EPTB (67, 68). A case was reported in which immunosuppressant therapy given for SLE in a patient coinfected with disseminated TB led to respiratory failure (69). Meningitis TB treatment is challenging because of the poor penetration of drugs (e.g., rifampin and streptomycin) into the CSF due to the impervious blood–brain barrier (70). EPTB is curable with ATT drugs only to an extent and may result in several complications; for example, patients on ATT treatment may develop acute kidney injuries and increase the risk for nephrotoxicity neuropathy and CNS toxicity (71–73). EPTB treatment also has some exclusion criterion; i.e., chemotherapy is detrimental during the first trimester of pregnancy as it prompts pregnancy termination. Specific adjuvant therapy, chemotherapy, and major surgery are suggested in some uncommon types of EPTB to avoid the complications of TB dissemination (**Figure 2**). Chemotherapy is required for genitourinary TB with surgery being substantial and reconstructive surgery required to repair the ureteral strictures (3).

The treatment of FGTB faces formidable challenges from coinfection (HIV, etc.); drug toxicity; obstetric, perioperative, and postoperative complications; reactivation; and emergence of drug-resistant bacteria (**Figure 2**). FGTB and HIV coinfection make the most deadly combination and is the leading cause of maternal mortality. Moreover, reactivation of bacilli has been observed in FGTB and HIV coinfection (12). HIV-induced immunosuppression in FGTB patients may also cause PID (74). ATT drugs can cause several complications in FGTB (41). Stem cells, nanotechnology, and colostrum are being used as a regenerative therapy to treat damaged endometrium, fallopian tubes, and ovaries (41). Vitamin D plays a crucial role in the treatment of FGTB (75). The use of steroids and immunotherapy is observed to a large extent among infertile patients and leads to resurgence of *M. tuberculosis* (76). Surgery in FGTB is performed as an adjunctive therapy during persistent or recurrent infection, the presence of nonhealing fistulae, and for multi-drug-resistant TB; however, reactivation of bacilli has been observed during surgery and has been detected after hysterosalpingography, laparoscopy, hysteroscopy, and laparotomy (77). Obstetric complications, such as preterm labor, increased rate of abortions, and neonatal mortality is high in FGTB. Perioperative complications, such as extreme hemorrhage with huge risk of damage to the pelvic and abdominal organs and the bowel, have been discerned during laparotomy (41). FGTB with pervasive adhesions in the uterus and blocked tubes and pelvis is not treatable even after successful treatment (41). Hysteroscopy is used to diagnose the adhesions and Asherman syndrome (78); however, it is associated with several complications in FGTB, such as, inability to visualize the cavity, excessive bleeding, perforation, bowel injury, peritonitis, and flare-up of genital TB (51, 79). Postoperative complications, such as bowel fistula and mortality rate are high in FGTB. Repeated invasive measures are required after ATT treatment for proper prognosis for fertility. The conception rate after ATT is only 12.8%, and the outcome of pregnancy could still be a live birth, spontaneous abortion, or ectopic pregnancy (80, 81). Furthermore, if patients are considered cured, their chances of pregnancy drop due to the irreversible damage of the fallopian tube and endometrium. Moreover, FGTB, if not properly treated, can cause permanent sterility through endometrial destruction and tubal damage (41). In vitro fertilization (IVF) is considered to be the successful modality for pregnancy in FGTB patients; however, a pregnancy rate of only 17.3% is observed even after successful treatment (82, 83).

The emergence of drug resistance among EPTB, particularly FGTB patients, is on the rise, and it poses a further threat to TB control. EPTB patients have a higher proportion of drug resistance compared to PTB patients (84). Furthermore, a high proportion of drug resistance is witnessed among the treatment failure cases of EPTB (52.7%) and PTB (48.1%) (85). The emergence of a multi-drug-resistant strain has been reported in FGTB (57). Engineered bacteriophages (Muddy, BPs33ΔHTH-HRM10, and ZoeJΔ45) are used as an adjunctive therapy against drug-resistant disseminated *Mycobacterium abscessus* (86). Antitubercular peptides, such as cathelicidins, defensins, granulysin, and hepcidin, are developed as novel TB therapeutics against drug-resistant TB (87).

## Genital Tuberculosis: Adeptness in Immune Modulation

**Figure 3:**
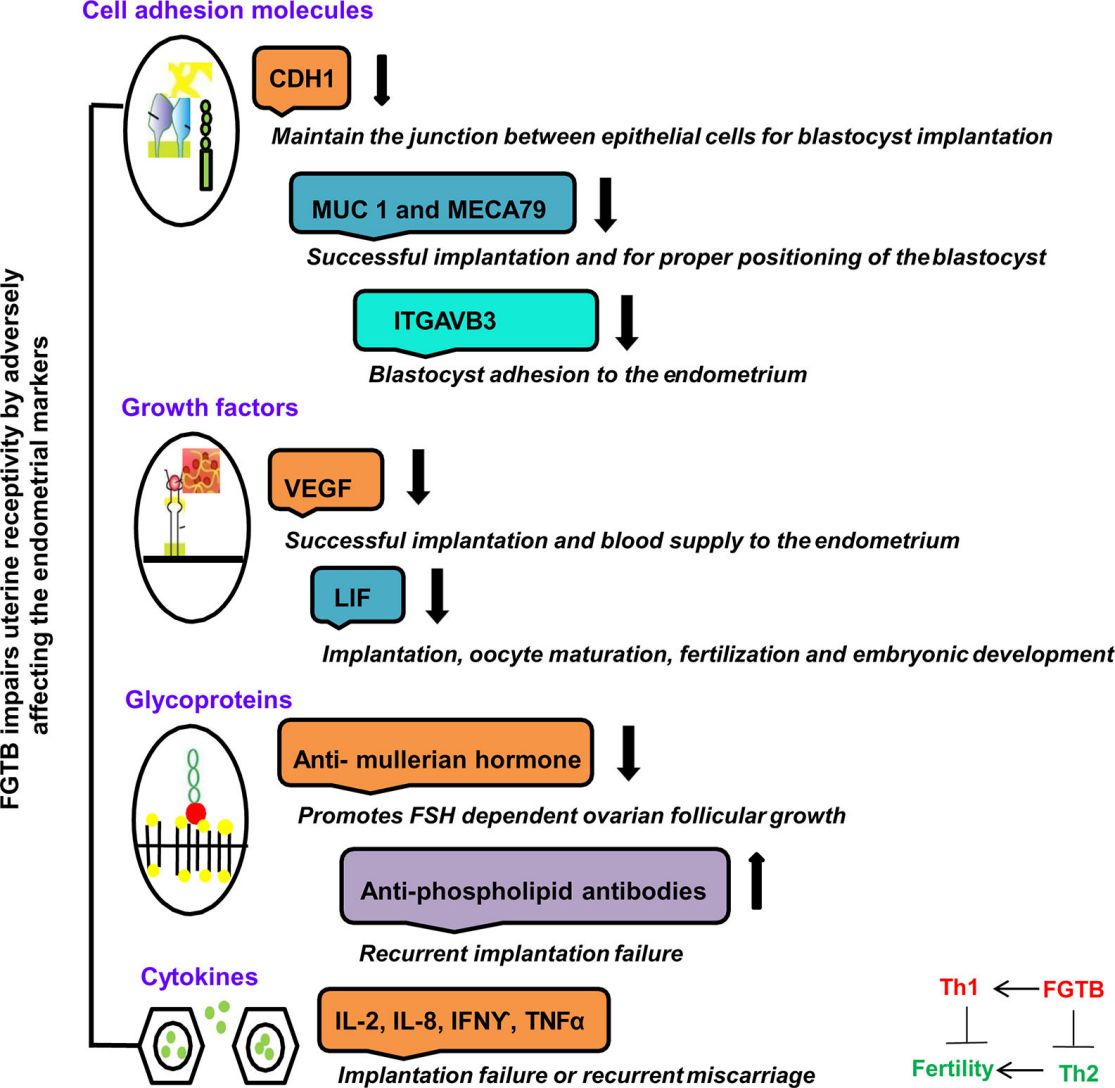
FGTB: Immune dysregulation compromises female fertility. The impact of FGTB on female fertility is depicted. FGTB adversely affects uterine receptivity through immune dysregulation. Various cell adhesion molecules, growth factors, glycoproteins, and cytokines mentioned here are potential biomarkers of uterine receptivity and for successful placentation. FGTB lowers the level of CDH1, MUC1, MECA79, and ITGAVB3, leading to recurrent implantation failure. Similarly, FGTB pares down the levels of VEGF and LIF, which are required for successful placentation, thus creating an unfavorable environment for embryonic implantation. Glycoproteins and cytokines are also required for embryonic development. FGTB also affects embryonic development through upregulating proinflammatory cytokine expression and antiphospholipid antibodies as well as by lowering anti-inflammatory cytokine expression and ovarian reserve markers, such as the antimullerian hormone.

Various molecules that are essential for implantation are being identified as potential players of uterine receptivity, such as growth factors, i.e., VEGF; cytokines, i.e., leukemia inhibitory factor (LIF) (88, 89); and cell adhesion molecules, i.e., CDH1 (E cadherin), ITGAVB3 (αvβ3), MUC-1 (Mucin-1), and MECA79, as well as hormones expressed during implantation (90, 91) (**Figure 3**). FGTB infection is found to alter the endometrial milieu and, thus, the UR, by causing immune modulation, endocrine disruption, activation of antiphospholipids antibodies, and microthrombosis, which leads to RIF, a major cause of infertility (92). FGTB significantly alters the level of ITGAVB3, MECA79, CDH1, MUC-1, and VEGF, leading to RIF (90). ITGAVB3 is essential for implantation, and its expression is reduced in both FGTB and unexplained recurrent pregnancy loss (90, 91). Additionally, an aberrant (reduced) expression of LIF has been reported in the endometrium in FGTB. The concentration of LIF is higher in fertile women compared to infertile females (93). LIF can activate signal transducers and activators of transcription 3 (STAT3) through a signaling cascade mechanism, which regulates UR and is further required for the transcription of VEGF, an angiogenic factor whose role during pregnancy is well studied (88, 90, 94, 95). FGTB lowers VEGF expression; thus creating an unfavorable environment for embryonic implantation (90). On the contrary, high VEGF levels contribute to the pathogenesis of EPTB; therefore, anti-VEGF agents are used in TB to prevent bacterial dissemination (96, 97). TB bacilli show an antigonadotropic effect in FGTB, impeding the production of progesterone and human chorionic gonadotropin (98). In FGTB, luteinizing hormone (LH) and follicle stimulating hormone (FSH) levels are high, and inhibin levels are very low (99). Inhibin is considered to be a more sensitive marker of ovarian reserve in FGTB compared to FSH (99, 100). Latent FGTB not only interferes with implantation in the basal endometrial layer, but also lowers the level of two ovarian markers, i.e., antimullerian hormone and antral follicle count (101). Furthermore, it has been observed that FGTB lowers the oocyte yield and the ovarian reserve (101).

Cytokine production differs in PTB and EPTB patients; females with normal pregnancy have been observed to have Th2-type cytokine milieu, whereas there has been shown to be an increased production of Th1-type cytokines in unexplained recurrent abortions (102, 103). The inflammatory environment in the endometrium prompts the preponderance of adverse cytokines and antibodies of the Th1 repertoire, making it nonreceptive to the embryo, thereby causing an implantation failure (92). However, T regulatory cells, a subset of CD4^+^ T cells limit the adaptive immune response and contribute to the persistence of chronic infection. Immune dysregulation has been reported in patients who have a past or present history of EPTB as observed by an increased production of T regulatory cells, high levels of IL-17, and CD4^+^ lymphocyte activation (104, 105).

## NRs and FGTB: Potential Markers and Drug Targets

**Figure 4:**
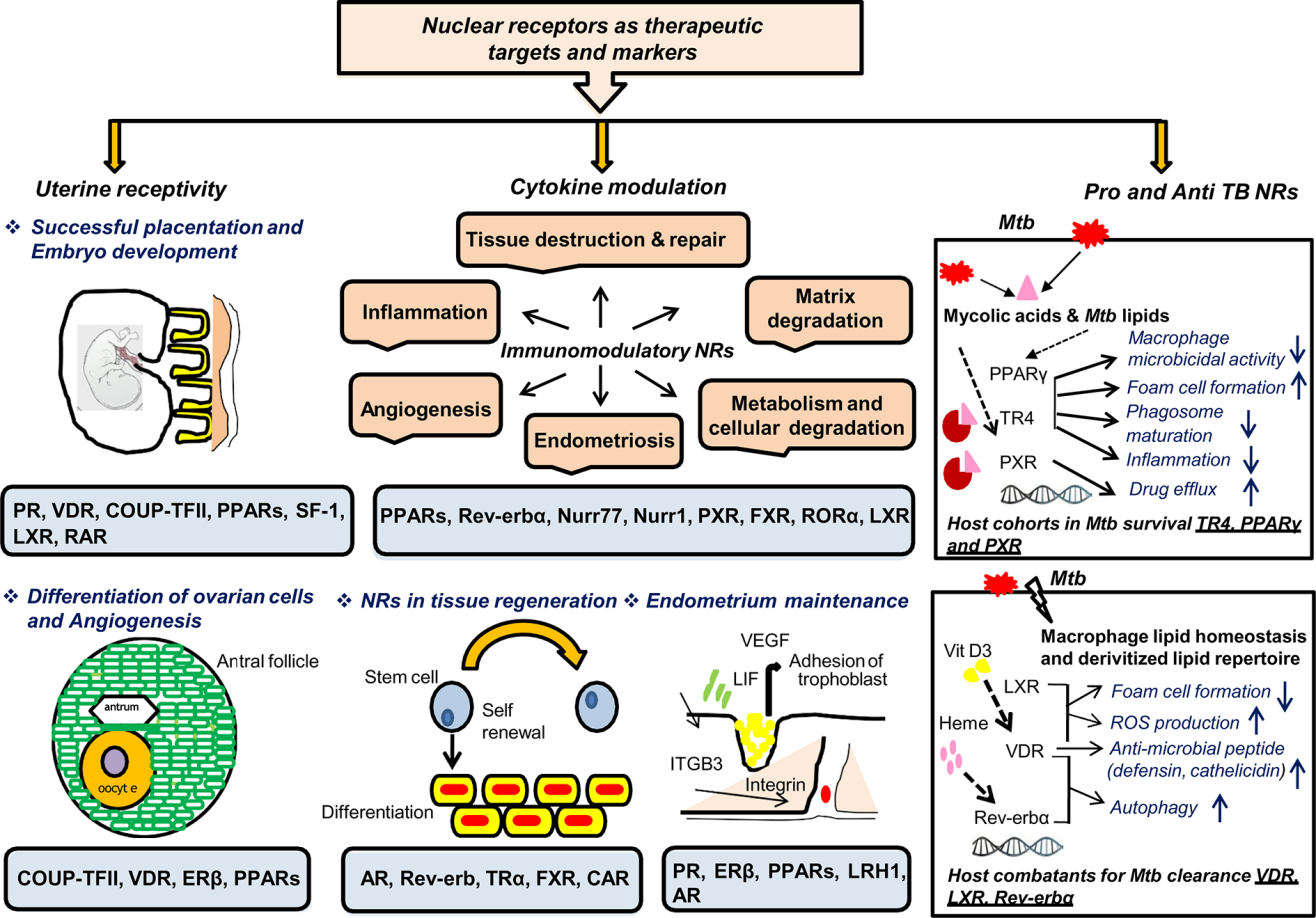
NRs are potential therapeutic targets and markers. NRs have many roles in TB, which makes them potential therapeutic targets for combating FGTB. NRs have been reported in female fertility; for example PR, VDR, COUP-TF, PPARs, SF-1, and LXR are essential for maintaining uterine receptivity through successful placentation and embryonic development. NRs such as COUP-TF, VDR, ERβ, and PPARs play an important role in differentiation of ovarian cells and angiogenesis. NRs such as PR, ERβ, PPARs, LRH1, and AR are reported in endometrium maintenance. NRs are also good immuno-modulators that may act either directly to combat the compromised tissue’s regenerative capacity or indirectly *via* CM to repair damaged tissues. NRs such as AR, Rev-erb, TRα, FXR, and CAR are reported for tissue regeneration, whereas PPARs, Rev-erbα, Nurr77, Nurr1, PXR, FXR, RORα, and LXR are known to modulate different cytokines’ milieu. Additionally, NRs enhance the self-renewing and differentiation capacity of transcription factors through direct modulation. NRs should be considered as TB biomarkers owing to their reported roles in both therapeutics and pathogenesis. NRs such as TR4, PPARγ, and PXR are considered as host cohorts in *M*. *tuberculosis* survival. Conversely, NRs such as VDR, LXR, and Rev-erbα are considered as good host combatants for *M. tuberculosis* clearance.

This review aims to accentuate three major points: (i) the diagnostic and treatment challenges of EPTB, particularly FGTB; (ii) the need for new therapeutics and diagnostics of EPTB, particularly FGTB; and (iii) the demand for FGTB biomarkers as a point-of-care diagnostic. NRs appear to be major potential therapeutic targets owing to their roles being reported as both pro-TB and anti-TB (**Figure 4**). NRs are ligand-activated transcriptional factors that act as molecular switches and can govern many physiological processes, such as metabolism, reproduction, and development. The superfamily of NRs shares a common structure containing an amino terminal domain, a conserved DNA-binding domain (DBD), a hinge region, and a ligand-binding domain (LBD) at the carboxy terminal. The amino terminal domain includes the activator function-1 region (AF-1), which interacts with several coregulatory proteins and is also a site for various posttranslational modification. The DBD is conserved and has two subdomains (for DNA binding and receptor dimerization), each containing 4 cysteine residues that coordinate with a zinc ion to form zinc finger motif. The hinge region consists of a nuclear localization signal, and the LBD harbors another activation function domain (AF-2) that can interact directly with coregulator proteins (106). NRs can exist as monomer, homodimer, and heterodimer that recognize a specific DNA sequence on the target genes known as response elements. NRs are classified into three categories based on the ligand variability: class I constitutes the endocrine receptors, class II includes orphan receptors, and class III comprises adopted orphan receptors. The endocrine receptors recognize steroid molecules and vitamins as their ligands and possess a high affinity toward them. The orphan receptors are those for which no endogenous ligand has been deciphered, and the adopted orphans are those whose ligands have been recently identified, and they bind to low-affinity dietary lipids. As various biological processes are regulated by NRs, pharmacological inhibition or dysregulation of them can lead to various diseases, including cancer, metabolic disorders, infertility, and neurodegeneration. They also play a significant role in infectious disease biology as many pathogens, for their own advantage, can modulate NRs either by interfering with their transcriptional activity or by changing their function. NRs have been studied in macrophage response to infectious disease, which also shows the potential role of NRs in combating infectious disease (107). Our earlier studies show a heterologous and noncanonical ligand receptor pairing, which clearly demonstrates that *M. tuberculosis* engage NRs (108–110). NRs, such as testicular receptor (TR4), peroxisome proliferator activated receptor (PPARγ), and pregnane X receptor (PXR), enhance *M. tuberculosis* survival by subverting the host innate immune defense mechanism and may increase the risk of dissemination (108, 109, 111). Our group has shown that *M. tuberculosis* cell wall lipids can crosstalk with NRs, such as PPARγ, TR4, and PXR. These NRs are involved in the formation of lipid-enriched foamy macrophages inside the host cell, which further enhances *M. tuberculosis* survival and subverts the immune response by abrogating the phagolysosomal fusion, inhibiting the secretion of proinflammatory cytokines and abating apoptosis. Furthermore, our group also reports that PXR causes TB drug nonresponsiveness in human macrophages by virtue of modulating drug efflux transporters (111). It has been observed that knockout of PPARγ in a mouse model reduces the growth of *M. tuberculosis*, lowers granulomatous infiltration, and enhances secretion of the proinflammatory cytokines (112). Moreover, NRs, such as vitamin D receptor (VDR) (113), Rev-erbα (114), and liver X receptor (LXR) (115), help with *M. tuberculosis* clearance. Interestingly, EPTB patients with multidrug-resistant TB have lower vitamin D levels (116). TR4 is identified as a marker for early TB detection in rhesus macaques, demonstrating that NRs are likely to make good biomarkers for TB (117). The expression level of TR4 is linked with severity of disease progression in the PBMCs of *M. tuberculosis*–infected rhesus macaques. Correspondingly, NRs can be modulated by small molecules, which allows them to be a potential therapeutic drug target. NRs also may have a role in EPTB, particularly FGTB which needs to be addressed further.

The three chief challenges pertaining to FGTB are UR, ER, and CM. These three factors are required for maintaining female fertility; their dysregulation, either directly or indirectly, leads to fertility issues. FGTB, either directly or indirectly, modulates UR and ER or CM, respectively; thereby, causing RIF. As mentioned before, NRs also play multifarious roles in female reproduction and in sustaining viable pregnancies (**Table 3**). Any perturbations in the expression of NRs could lead to spontaneous abortions. NRs, such as liver receptor homolog 1 (LRH1), retinoic acid receptor (RAR), chicken ovalbumin upstream promoter (COUP-TFII), steroidogenic factor (SF-1), androgen receptor (AR), LXR, VDR, progesterone receptor (PR), estrogen receptor (ERβ), and PPARs have been reported for successful uterine implantation and endometrium maintenance. VDR has also been reported to be important for the differentiation of granulosa cells. The NR LRH1 is reported to be important for mouse fertility (118), ovulation, and ovarian steroidogenesis (119, 120). RAR is involved in early embryonic development (121). COUP-TFII is required for placental development and angiogenesis (122, 123). SF-1 is reported for folliculogenesis and in the process of ovulation with its absence in granulosa cells leading to impaired ovulation (124, 125). AR signaling is essential for endometrial function, whereas its perturbation leads to reproductive failure (126). LXR modulates ovarian endocrine and exocrine function and uterus contractility (127). VDR expression increases during pregnancy and helps with reproductive function (128). Vitamin D has roles in folliculogenesis, differentiation, luteinization, and steroidogenesis as well as altering antimullerian hormone signaling and progesterone production (129). Vitamin D deficiency in pregnancy increases the fortuity of preterm birth and preeclampsia (130, 131). PR signaling is essential for the initiation and maintenance of pregnancy (132). ERβ is essential for maintaining the endometrium quiescence and vasculature (133). PPARs are essential for trophoblast invasion, decidualization, tissue remodeling, ovarian function, and placental formation (134–136). Additionally, circadian rhythm disturbance is reported to affect female fertility (137). Rev-erb is a circadian NR, which maintains the circadian rhythm (138) and may have a role in female fertility. Because NRs play crucial roles in female reproduction, they could make good therapeutic targets to combat female infertility.

**Table 3:** Role of Nuclear receptors in female reproduction.

Nuclear Receptors	Functions in Female Reproduction
LRH1	Essential for ovarian steroidogenesis and ovulation
PR	Implantation, decidualization, and preventing endometriosis
ERα	Endometriosis progression
ERβ	Maintenance of endometrium quiescence and vasculature
SF-1	Development of reproductive tissue, ovulation, and folliculogenesis
VDR	Differentiation of granulosa cells, folliculogenesis, luteinization, and steroidogenesis
PPARα	Proliferation and differentiation of ovarian cells
PPARβ	Implantation and decidualization
PPARγ	Trophoblast invasion and placental formation, decidualization, and preventing endometriosis
AR	Maintenance of endometrium physiology
RAR	Embryonic development, growth, and reproduction
Rev-erb	Regulating the circadian rhythm
COUP-TFII	Placental development and angiogenesis
LXR	Control ovarian endocrine and exocrine function and uterine contractility

RIF occurs due to compromised ER capacity; therefore, stem cell therapy for ER could be helpful. Many NRs have gained attention in stem cell biology (139–142); estrogen receptor (ERα), PR, and PPARγ are all implicated in endometriosis (143–146); and AR, thyroid receptor (TR), farnesoid X receptor (FXR), Rev-erb, and constitutive androstane receptor (CAR) are reported in tissue regeneration (147–152). Female fertility is compromised due to endometriosis. NRs are known to modulate endometriosis; for example, loss of PR expression leads to endometriotic tissue becoming resistant to progesterone, leading to endometriosis (146). PR helps to relieve pain in endometriosis by limiting inflammation and the growth of endometriotic tissue PPARs and retinoid X receptor alpha are expressed in abortive trophoblastic tissue and are upregulated in extra villous trophoblast in recurrent miscarriages (153, 154).

*M. tuberculosis* modulates various cytokines’ milieu, such as interferon γ (IFNγ) and interleukin- (IL2) in the endometrium and TNFα, IL-6, IL-4, and IL-8 in the blood (155, 156). Additionally, administration of IFNγ, TNFα, and IL-2 is reported to cause abortions in pregnant mice (102, 157, 158). Moreover, IL-1β is shown to promote endometriosis and angiogenesis (159, 160). Conversely, IL-6 and IL-10 are reported to have increased production in normal pregnancy compared to spontaneous abortion (103). Various proinflammatory, anti-inflammatory, and pleiotropic NRs, such as retinoic acid receptor-related orphan receptor, nuclear receptor related (Nurr) 77, Nurr1, RORα, PXR, FXR, PPARα, LXR, Rev-erbα, and PPARs, are known as immune modulators as they can modulate different cytokines’ milieu (114, 161–167). LXR is known to inhibit proinflammatory cytokine expression and is also responsible for maternal-fetal cholesterol transport; there is also a reduction in LXR expression in miscarriages (168, 169). Additionally, FGTB modulates pregnancy-related hormones, such as human chorionic gonadotropin and progesterone, which are known to function *via* their cognate endocrine receptors (98). Taken together, NRs seem to be a promising target to combat FGTB by addressing the issues of UR, ER, and CM. Extensive knowledge about the expression and function of the NRs in FGTB is lacking and needs to be addressed.

FGTB modulates the localized endometrial immune repertoire, which has been reported to modulate UR. There are various reports illustrating the function of the endometrial immune repertoire in recurrent spontaneous miscarriage (170), polycystic ovarian syndrome (171), endometriosis (172), and unexplained infertility (173). Given the reported role of NRs in the regulation of uterine implantation and CM as well as being cognate to pregnancy-related hormones, for example, estrogen, progesterone, human chorionic gonadotropin, and human placental lactogen, which function *via* estrogen receptor, PR, and VDR, respectively (174–178), they are good potential targets to alleviate the disease. NRs could be excellent host-directed targets in FGTB as evident from previous reports of NRs in TB. *M. tuberculosis* can modulate the expression of NRs by certain crosstalk with its lipid repertoire. It would be interesting to see whether *M. tuberculosis* components interfere or modulate the interaction of pregnancy-related hormones with their cognate endocrine receptors. It would be interesting to decipher whether the *M. tuberculosis* components also relay their effect through orphan or adopted orphan receptors.

## Discussion

Globally, EPTB and, in particular, FGTB are growing problems with increasing rates of morbidity and mortality worldwide. FGTB represents the most perilous form of EPTB and is the leading cause of infertility and recurrent implantation failure in females. FGTB cases are asymptomatic in early stages, and untreated FGTB can cause permanent sterility through endometrial destruction and tubal damage. FGTB diagnosis is arduous because of varied clinical presentations, misdiagnosis, associated comorbidities, arduousness in acquiring of clinical samples, poor sensitivity, it is often asymptomatic and paucibacillary, emergence of drug resistance, lack of point of care, impenetrable sites, and abstruse positioning of the organs. Likewise, the treatment of FGTB faces formidable challenges due to drug toxicity; HIV coinfection; obstetric, perioperative, and postoperative complications; reactivation; and emergence of drug-resistant bacteria. Our review rolls out the possible remedies to prevent FGTB by precluding several of these challenges and also highlights the need for exaction of biomarkers in FGTB.

It is imperative to understand that FGTB adversely affects UR and causes immune modulation, which promptly leads to abortions and also reduces the chances of conception. We emphasize the imperative mechanism of FGTB-associated female infertility by highlighting the three major challenges, i.e., UR, ER, and CM. FGTB adversely affects various endocrine hormones (progesterone, estrogen, and human chorionic gonadotropin), cytokines, growth factors (LIF and VEGF), and cell adhesion molecules (ITGAVB3, MECA79, CDH1 and MUC-1), which are responsible for the maintenance of successful pregnancy. We epitomize the need to identify the molecular switches at the interface of FGTB and mechanisms associated with female infertility.

Given the above challenges in FGTB, there is an exigent need to identify FGTB-specific biomarkers from accessible samples. NRs have been reported as both pro- and anti-TB but have gained less attention in FGTB. They are reported to modulate female fertility and stem cell plasticity and are also known as immune modulators. We attempt to invoke interest in the exploration of NRs as a novel therapeutic target in FGTB-associated female infertility and as a potential biomarker. NRs, which are cognate to pregnancy-related hormones (estrogen, progesterone, and human chorionic gonadotropin) and have been cited in female reproduction and regeneration, prompt us to postulate them as a potential player and target to combat FGTB-associated female infertility by addressing the issues of UR, ER, and CM.

The topic is of immediate importance because of the abrupt increase in disease severity, drug resistance, and lack of a knowledge base of the major diagnostic and treatment challenges, which leads to exacerbation in FGTB. Although a large number of biosignatures and mechanisms have been reported in FGTB, there is a paucity of specific targets and biomarkers. Our review provides the conceptual advance; it postulates the role of NRs as a potential target and biomarker in FGTB. The description is comprehensive and is factual. Fostering innovative research is required to (i) develop highly permeable, safe, and nontoxic drugs with a novel mechanism of action and target; (ii) identify biomarkers and point-of-care diagnostics; and (iii) develop a strategy to shorten the treatment regimens and reduce treatment-related functional disability.

## Author Contributions 

SG and PG designed the study. SG and PG wrote the review. PG contributed to the overall supervision of the manuscript. All authors contributed to the article and approved the submitted version.

## Funding

This work was supported by the Council of Scientific and Industrial Research (CSIR) RC project (OLP115) to PG. This work is also supported by the Department of Biotechnology, Ministry of Science and Technology, National Bioscience Award project (GAP-0162) to PG. We thank IMTECH, a CSIR laboratory, for the facilities and financial support. The funders had no role in study design, data collection, or interpretation or in any decision to submit the work for publication.

## Conflict of Interest

The authors declare that the research was conducted in the absence of any commercial or financial relationships that could be construed as a potential conflict of interest.

## Abbreviations

EPTB, Extrapulmonary Tuberculosis; PTB, Pulmonary Tuberculosis; TB, Tuberculosis; FGTB, Female Genital Tuberculosis; RIF, Recurrent Implantation Failure; UR, Uterine Receptivity; ER, Endometrium regeneration; CM, Cytokine modulation; LIF, Leukemia inhibitory factor; VEGF, Vascular endothelial growth factor; NR, Nuclear Receptors; TR4, Testicular receptor; PPAR, Peroxisome Proliferator Activated Receptor; PXR, Pregnane X Receptor; VDR, Vitamin D Receptor; LXR, Liver X Receptor; PR, Progesterone receptor; LRH1, Liver receptor homolog 1; Nurr, Nuclear receptor related; AR, Androgen receptor; COUP-TF, Chicken Ovalbumin Upstream Promoter; SF-1, Steroidogenic Factor; FXR, Farnesoid X Receptor; IL, Interleukin.
